# Ecological association between a deprivation index and mortality in France over the period 1997 – 2001: variations with spatial scale, degree of urbanicity, age, gender and cause of death

**DOI:** 10.1186/1471-2458-9-33

**Published:** 2009-01-22

**Authors:** Grégoire Rey, Eric Jougla, Anne Fouillet, Denis Hémon

**Affiliations:** 1 INSERM, CépiDc, IFR69, Le Vésinet, France; 2 Institute of Public Health Surveillance (InVS), Saint-Maurice, France; 3 INSERM, U754, Université Paris Sud 11, IFR69, Villejuif, France

## Abstract

**Background:**

Spatial health inequalities have often been analysed in terms of deprivation. The aim of this study was to create an ecological deprivation index and evaluate its association with mortality over the entire mainland France territory. More specifically, the variations with the degree of urbanicity, spatial scale, age, gender and cause of death, which influence the association between mortality and deprivation, have been described.

**Methods:**

The deprivation index, 'FDep99', was developed at the '*commune*'(smallest administrative unit in France) level as the first component of a principal component analysis of four socioeconomic variables.

Proxies of the Carstairs and Townsend indices were calculated for comparison.

The spatial association between FDep99 and mortality was studied using five different spatial scales, and by degree of urbanicity (five urban unit categories), age, gender and cause of death, over the period 1997–2001.

'Avoidable' causes of death were also considered for subjects aged less than 65 years. They were defined as causes related to risk behaviour and primary prevention (alcohol, smoking, accidents).

**Results:**

The association between the FDep99 index and mortality was positive and quasi-log-linear, for all geographic scales. The standardized mortality ratio (SMR) was 24% higher for the *communes *of the most deprived quintile than for those of the least deprived quintile. The between-urban unit category and between-*région *heterogeneities of the log-linear associations were not statistically significant. The association was positive for all the categories studied and was significantly greater for subjects aged less than 65 years, for men, and for 'avoidable' mortality.

The amplitude and regularity of the associations between mortality and the Townsend and Carstairs indices were lower.

**Conclusion:**

The deprivation index proposed reflects a major part of spatial socioeconomic heterogeneity, in a homogeneous manner over the whole country. The index may be routinely used by healthcare authorities to observe, analyse, and manage spatial health inequalities.

## Background

Deprivation indices are commonly used to describe spatial health heterogeneity. Initially defined by Townsend as a "state of observable and demonstrable disadvantage relative to the local community or the wider society to which an individual, family or group belongs"[1], deprivation has been quantified using a score obtained by summing standardized variables, each measuring different ecological dimensions derived from census data. Income data was not included in the calculation of the Townsend index, mainly because it was not possible to obtain those data in the United Kingdom when the index was designed. Nevertheless, the concept of deprivation was not defined as a proxy of income, but as an accumulation of disadvantages.

Other deprivation indices have been developed on that basis [2-10]. The most recent are generally calculated by factor analyses [11], and include income data [6,7,12-14]. Thus, the weights allocated to each dimension aim to reflect optimally the variability of the socioeconomic dimensions considered within the population studied. This choice implicitly supposes that the probably high correlation between the various components prevents determining which components are the most relevant and their weightings.

One criticism of the most classic indices used, the Carstairs and Townsend indices, [8,15-24] relates to their urban view of deprivation, explaining the weaker associations between those indices and the health indicators observed for rural areas, compared to those for urban areas [10,21,23-27]. For instance, the meaning of the proportion of households without a car, which is a variable used to define those indices, differs depending on whether rural or urban areas are considered.

Health inequalities are a quite recent subject in France. Previous studies were essentially based, at an individual level, on specific surveys [28,29], and, at an ecological level, on specific regions of France [7,30]. To the authors' knowledge, no spatially defined deprivation index has yet been proposed for analysis of health inequalities on a routine basis for all of France.

The association between socioeconomic level and mortality at individual or population level has been widely studied [31,32] and a strong association between deprivation and mortality has been reported [5,6,10,12,13,15,17,18,20,21,33-37].

Some characteristics of the spatial association have yet to be specified.

First, it is important to evaluate the extent to which the choice of the spatial scale influences the association between deprivation and mortality. This has rarely been done [8,38,39].

Secondly, the rural-urban gradient is one of the major influential factors in spatial issues. Over the whole of France, the homogeneity of the association between deprivation and mortality with respect to the rural-urban gradient and various regions had not yet been studied.

Lastly, among the mechanisms explaining socioeconomic mortality differences, the risk behaviours targeted by primary prevention (smoking, alcohol, road traffic) have often been mentioned [35,40,41]. The association between deprivation and 'avoidable' mortality may be analysed by specifically considering the causes of death that are the most frequently linked with risk behaviours for subjects aged under 65 years [20,41].

The aim of the present study is to propose a deprivation index, based on the census and the tax authority's household income data, which may be used in both rural and urban contexts, and to evaluate the ecological association between that index and mortality. More specifically, the spatial determinants influencing the association have been addressed and the variations with age, gender and cause of death described, with a specific focus on the causes of 'avoidable' mortality.

## Methods

### Spatial scale

Mainland France has a population of nearly 60 million. Five administrative or statistical spatial units, of different sizes, were considered: *communes *(the smallest administrative units in France, 30,500 units), *cantons *(3,700 units), *zones d'emploi *(350 units), *départements *(96 units) and *régions *(22 units). The main results were calculated on the *commune *scale, with a median population of 500 and a wide range of variation, from 80 to 400,000.

### Urban unit category (UUC)

The urban unit concept developed by the National Institute for Statistics and Economic Studies (INSEE) was used to define the degree of urbanicity of the *communes*. An urban unit is a group of *communes *in which no residence is separated from the next by more than 200 metres. There are five urban unit categories (UUC) of *commune*: rural (less than 2,000 people), quasi-rural (population from 2,000 to 9,999), quasi-urban (population from 10,000 to 99,999), urban (population from 100,000 to 1,999,999) and Paris-and-suburbs (Paris Urban Unit).

### Deprivation index

The deprivation index, 'FDep99', was constructed on the *commune *scale, using the socioeconomic data available on that scale: the 1999 population census (source: INSEE) and the tax authority's 2001 household income data (source: INSEE). As the latter data were only available for *communes *of more than 50 households, the analyses were limited to 30,500 of the 36,600 *communes*, representing 99.2% of the whole population. The index was constructed in order to ensure the following properties: one-dimensional; maximization of the heterogeneity of the components; and consistent association with the components.

Four variables derived from previous studies [5-7,9,12], each representing a fundamental dimension of socioeconomic level, of homogeneous meaning over the whole country, and correlated with the other variables within and between the UUC, were selected: the median household income, the percentage high school graduates in the population aged 15 years and older, the percentage blue-collar workers in the active population, and the unemployment rate. The first two variables constitute negative dimensions of deprivation, and the last two constitute positive dimensions.

FDep99 was defined as the first component of a principal component analysis (PCA) of those four variables. FDep99 accounted for 68% of the total variation and was strongly correlated with each of the components in a manner consistent with deprivation (negatively with income and the percentage high school graduates, and positively with the percentage blue-collar workers and the unemployment rate). The PCA coefficients for the four variables were very similar when calculated separately for each UUC and for all of France.

Proxies of the Townsend and Carstairs indices were calculated in order to compare them with the present index. Originally, the Townsend index was generated from British data as the sum of the following standardized variables: percentage of unemployed people in the active population, percentage of not-owner-occupied households, percentage of households without a car and percentage of overcrowded households. The original Carstairs index was calculated, using the same method, but the percentage of not-owner-occupied households was replaced by the percentage low social class people. In this study, the mean number of occupants per room and the percentage of blue-collar workers in the active population were used instead of the percentage overcrowded households and percentage low social class people, respectively, as the latter data were not available on a small scale in France.

For the units larger than the *commune*, the FDep99 index was calculated as the population-weighted mean of the *commune *values. As that measurement is only optimal for a normal distribution of FDep99, the population weighted median FDep99, a non parametric indicator, was also calculated. The latter was correlated (Pearson ρ = 0.99) with the population weighted mean on each spatial scale. Thus, only the results with population weighted means are shown.

### Mortality data

The mortality data were derived from the Inserm-CépiDc database for mainland France for the period 1997–2001. Overall there were 2,650,390 deaths. The commune of residence, which is systematically included in the death record, was used as the spatial location.

The underlying causes of death were analysed and classified using the 17 categories aggregated by Eurostat. An additional category, 'avoidable' causes linked to risk behaviours targeted by primary prevention [41], was defined for 'premature' deaths occurring before age 65 years only. This category consisted in causes of death related to smoking and alcohol consumption (lung, trachea and bronchus cancers (ICD10 Code: C32–C34), aerodigestive tract cancers (C00–C14), oesophagus cancer (C15), alcohol abuse (F10), chronic liver disease (K70, K73–K74)), drug dependence (F11), AIDS (B20–B24), transport accidents (V01–V99), suicides (X60–X84) and homicides (X85–Y09).

### Association measures

The standardized mortality ratio (SMR) was used to characterize the mortality differentials for the period 1997–2001. SMR was calculated as the ratio of the observed mortality in a spatial unit, over the corresponding expected mortality (national mortality rates for the period 1997–2001 applied to the spatial unit population, by age and gender). The association between the deprivation index and SMR was assessed using two measures:

- the ratio (SMR_Qi/_SMR_Q1_) between the SMR of the spatial unit whose deprivation index was between the (i-1)^th ^and the i^th ^quintile (SMR_Qi_: the SMR of the i^th ^deprivation quintile) and the SMR of the spatial unit whose deprivation index was below the first quintile (SMR_Q1_),

- the log-linear trend, defined as the linear association between the logarithm of the SMR and the deprivation index. Log-linear trend was considered rather than linear trend because of the multiplicative structure of the SMR. However, as every SMR considered in this article was close to 1, the SMR was close to log(SMR + 1). Therefore, the log-linear associations were considered approximately equal to the linear associations.

This measure quantified the average relative increase in mortality per unit of deprivation over the whole range of deprivation. As the observed association was quasi-linear, it was used to compare the amplitudes of the associations for different categories and to adjust mortality variations for deprivation. The association was calculated as "β", derived from the following Poisson model allowing for overdispersion:

$$\{\begin{array}{c} {\text{O}}_{\text{u}}\propto \text{P}(\lambda_{\text{u}})\\ \log(\lambda_{\text{u}})=\log({\text{E}}_{\text{u}})+\alpha +\beta \cdot \text{FDep}99_{\text{u}} \end{array},$$

In which 'u' is a spatial unit, 'O_u_' is the number of deaths observed in 'u' for the 1997–2001 period, 'E_u_' is the expected number of deaths in 'u', 'FDep99_u_' is the mean deprivation index in 'u', 'α' is the intercept and 'β' is the log-linear trend.

## Results

### Variations in mortality and deprivation indices with urban unit category (UUC)

The mortality was significantly lower, by at least 9%, in the Paris-and-suburbs UUC than in the other UUC (Table 1). UUC was a major variation factor for the three synthetic indices (FDep99, Townsend and Carstairs).

**Table 1:** Association between the various deprivation indices and all-cause mortality on the *commune *scale, by degree of urbanicity

			**Urban unit category (UUC)**				
			
		**All of France**	**Rural**	**Quasi-rural**	**Quasi-urban**	**Urban**	**Paris-and-suburbs**
Number of spatial units		30,498	24,533	2,466	1,774	1,312	414

Population (in thousand)		59,273	14,101	7,019	11,143	17,196	9,814

	SMR		1.13*	1.17*	1.13*	1.09*	1.00

**FDep99**	Mean	0	0.34	0.36	0.33	-0.03	-1.07
	Q5-Q1	2.73	1.92	2.03	1.85	2.46	3.54
	SMR_Q5_/SMR_Q1_	1.24*	1.11*	1.17*	1.20*	1.29*	1.30*
	Adjusted SMR		1.01	1.04	0.99	1.00	1.00 (ref.)

**Townsend**	Mean	0	-2.92	-1.43	0.15	1.29	2.94
	Q5-Q1	8.62	3.82	5.06	6.57	7.54	9.43
	SMR_Q5_/SMR_Q1_	1.02	1.09*	1.15*	1.12*	1.13*	1.11
	Adjusted SMR		1.24*	1.25*	1.16*	1.11*	1.00 (ref.)

**Carstairs**	Mean	0	-1.46	-0.53	0.22	0.59	1.28
	Q5-Q1	6.73	4.59	5.05	5.63	6.98	8.32
	SMR_Q5_/SMR_Q1_	1.12*	1.10*	1.16*	1.19*	1.25*	1.15*
	Adjusted SMR		1.19*	1.21*	1.15*	1.10*	1.00 (ref.)

FDep99: First component of a PCA on the *commune *scale of the variables: median income, % blue-collar workers, % high school graduates, and unemployment rate

Townsend and Carstairs: sum of standardized variables

Q5-Q1: Difference between the index average of *communes *whose index is higher than the fourth quintile and the index average of *communes *whose index is lower than the first quintile

SMR: Standardized Mortality Ratio for the period, 1997–2001

SMR_Q5_/SMR_Q1_: ratio of the SMR of *communes *whose deprivation is higher than the fourth quintile and SMR of the *communes *whose deprivation is lower than the first quintile

Adjusted SMR: SMR of the UUC (ref. 1 for Paris-and-suburbs) adjusted with a log-linear trend for deprivation;

*: the value is statistically significantly different from 1 at the 5% level

The averages of the three indices behaved differently across the UUC (Table 1): the FDep99 index was lower for urban categories than for rural categories, while the Townsend and Carstairs indices exhibited the opposite pattern. The differences between the average values of the upper and lower quintiles were markedly higher for the most urban UUC for each of the indices.

While the Townsend and FDep99 indices were weakly correlated (Pearson's r = +0.1) on the all-of-France scale, their correlations were greater than 0.5 within each UUC. The correlation of the FDep99 index with the Carstairs index was higher, 0.5 on the all-of-France scale, and close to 0.8 within each UUC. Similar results were obtained when Spearman non-parametric correlation coefficients were considered.

### Associations between deprivation and all-cause mortality, by index and degree of urbanicity

The association between mortality and the FDep99 index was strong and significant for all of mainland France and also within each UUC (Table 1). Considering the whole of France, the SMR was 24% higher in the fifth quintile *communes *than in the first quintile *communes; *those associations were weaker, or non-significant, with the Townsend and Carstairs indices. However, for each UUC, the associations between mortality and the Carstairs or Townsend index were positive, statistically significant, and of smaller amplitude than for the FDep99 index. The mortality differentials between the first and last quintiles were higher for the most urbanized UUC, as was the case for the deprivation differentials.

**Figure 1:**
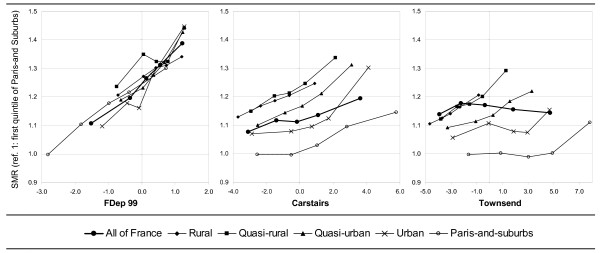
**SMR as a function of the various deprivation index quintiles, on the *commune *scale, by degree of urbanicity (UUC)**. SMR: standardized mortality ratio for the period 1997–2001. FDep99: First component of a PCA on the *commune *scale of the variables: median income, % blue-collar workers, % high school graduates, and unemployment rate. Townsend and Carstairs: sum of standardized variables.

The association between mortality and the three deprivation indices was close to linearity (equivalent to log-linearity as the relative risks were close to 1) in all the UUC considered separately (figure 1). The between-UUC heterogeneity of the log-linear trends associating the deprivation indices with the SMR (interactions between deprivation index and UUC) was not statistically significant for any of the three indices.

However, the between-UUC mortality differentials were very low, at a given level of FDep99, compared to the same differentials for a given level of the Carstairs or Townsend indices (figure 1): after adjustment for the FDep99 log-linear trend, the between-UUC heterogeneity of the SMR, calculated by including UUC as a categorical variable in the log-linear model, was not statistically significant. In contrast, for the Townsend and Carstairs indices, the between-UUC heterogeneity of the SMR remained statistically significant after the same adjustment (table 1).

With regard to the other results, only those for the FDep99 index have been presented.

### Geographic variations in FDep99 index and all-cause mortality

**Figure 2:**
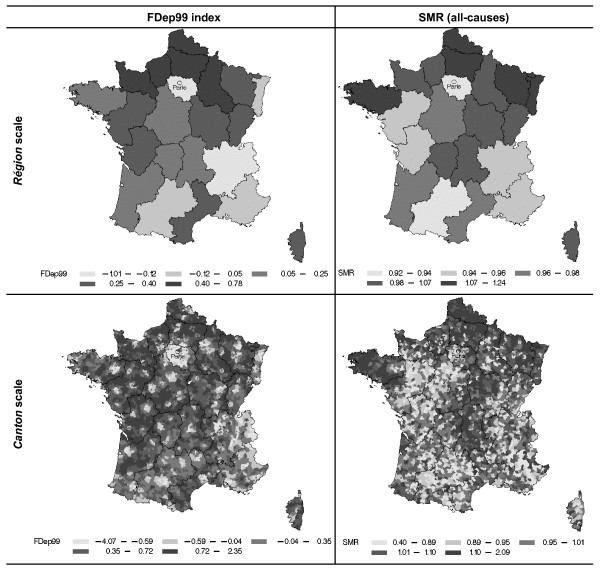
**Geographic distribution of the FDep99 index and all-cause mortality on the *region *and *canton *scales**. FDep99: First component of a PCA on the *commune *scale of the variables: median income, % blue-collar workers, % high school graduates, and unemployment rate. SMR: standardized mortality ratio for the period 1997–2001.

The geographic variations in FDep99 index and SMR for both large and small scales are illustrated by the maps on the *région *and *canton *scales (figure 2).

The *région*-scale distributions of mortality and FDep99 index were similar, showing a positive south-north gradient and low mortality in the Paris *région*. However, some differences were noteworthy, such as the extremes in the east and west of France, where mortality was high but the FDep99 was quite low.

On the *canton *scale, the similarities were less clear. The same main pattern was observed for mortality, while the FDep99 index distribution was influenced by strong local rural-urban variations. The less deprived spots consisted in urban areas (figure 2).

### Associations between the FDep99 index and all-cause mortality by scale

**Figure 3:**
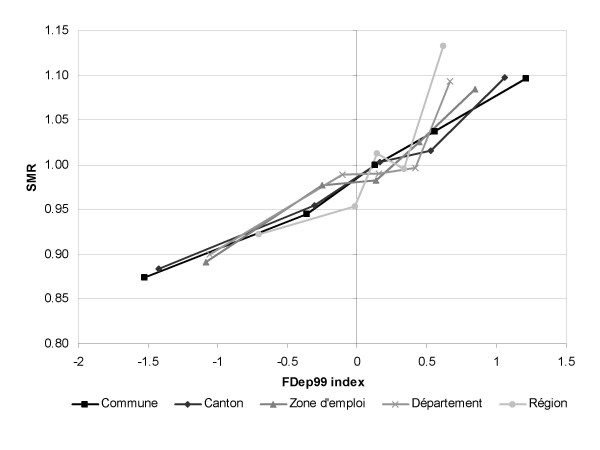
**SMR as a function of FDep99 index, by scale**. FDep99: First component of a PCA on the *commune *scale of the variables: median income, % blue-collar workers, % high school graduates, and unemployment rate. SMR: standardized mortality ratio for the period 1997–2001.

The association between the FDep99 index and mortality was positive for all the spatial scales considered (figure 3). However, the association was more regular and linear for the finest scales, *commune *and *canton*. The association on the *région *scale had a singular pattern, accentuating the highest mortality for the last quintile observed with all the scales.

It is noteworthy that when an adjustment for the higher levels was included in a model, the association on the finest scales remained significantly positive. The association between the FDep99 index and mortality on the *commune *scale was significantly positive within each *région*. The between-*région *heterogeneity of the log-linear trends (interactions between the FDep99 index and *régions*) was not statistically significant (p = 0.33).

Specific associations were evaluated using the finest scale (*commune*) on which the association was the strongest and most regular.

### Association between the FDep99 index and all-cause mortality by age and gender

**Figure 4:**
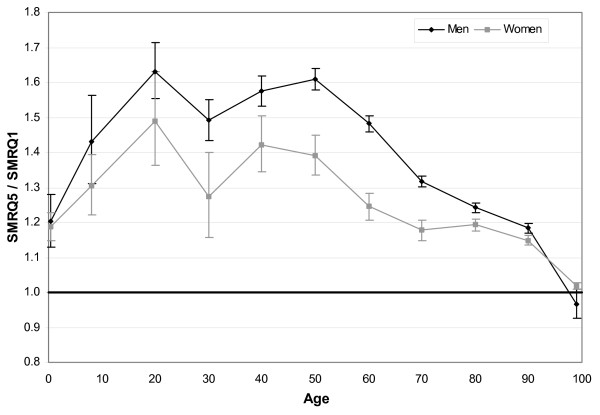
**All-cause mortality differentials with FDep99 index (SMRQ5/SMRQ1), by age and gender**. FDep99: First component of a PCA on the *commune *scale of the variables: median income, % blue-collar workers, % high school graduates, and unemployment rate. SMR: standardized mortality ratio for the period 1997–2001. SMRQ5/SMRQ1: ratio of the SMR of *communes *whose FDep99 index was higher than the fourth quintile and SMR of the *communes *whose FDep99 index was lower than the first quintile.

With the FDep99 index, the mortality differentials by *commune *varied strongly by age and gender (figure 4). For children aged less than 1 year and each gender, the mortality was 20% higher for the fifth FDep99 quintile than for the first. This differential was larger for subjects aged between 25 and 65 years, and to a lesser extent for the 25–34 and 55–64 age groups, with average mortality differences of 55% for males and 32% for females. The mortality differentials were lower for subjects aged 65 years and over, and statistically non-significant for subjects aged 95 and over.

Except for children aged less than 1 year and subjects aged 95 years and over, the mortality differentials were from 20% to 100% higher for men than for women.

The variations of mortality differentials, according to UUC and scale, were similar for both genders, with greater amplitude for men.

### Association between the FDep99 index and mortality by cause-of-death category

Except for infectious diseases and ill-defined conditions, mortality was significantly higher for the most deprived *communes *for all cause-of-death categories (Table 2). In terms of the FDep99 index, the categories with the highest mortality differentials were injury and poisoning, digestive system diseases, mental disorders, endocrine and nutritional diseases, pregnancy and childbirth diseases, respiratory diseases and cardiovascular diseases.

**Table 2:** Mortality differentials with the Fdep99 index (SMRQ5/SMRQ1) on the *commune *scale, by cause of death category and gender

		**SMRQ5/SMRQ1**					
		
	**% Total**	**All**		**Men**		**Women**	
1. Infectious diseases	1.7	1.01	[0.98; 1.04]	0.98	[0.94; 1.02]	1.05	[1.00; 1.09]
2. Neoplasms	27.9	1.16	[1.15; 1.17]	1.26	[1.25; 1.27]	1.02	[1.01; 1.03]
3. Blood diseases	0.5	1.19	[1.13; 1.26]	1.20	[1.11; 1.31]	1.19	[1.10; 1.28]
4. Endocrine and nutritional diseases	3.2	1.42	[1.39; 1.46]	1.46	[1.41; 1.51]	1.40	[1.36; 1.44]
5. Mental disorders	3.0	1.48	[1.45; 1.52]	1.68	[1.62; 1.74]	1.37	[1.33; 1.41]
6. Nervous system diseases	3.3	1.08	[1.06; 1.11]	1.14	[1.10; 1.18]	1.04	[1.01; 1.07]
7. Cardiovascular diseases	30.9	1.31	[1.30; 1.32]	1.36	[1.35; 1.38]	1.27	[1.26; 1.29]
8. Respiratory system diseases	7.5	1.34	[1.32; 1.36]	1.48	[1.45; 1.51]	1.22	[1.19; 1.24]
9. Digestive system diseases	4.7	1.48	[1.46; 1.51]	1.63	[1.59; 1.67]	1.34	[1.30; 1.38]
10. Skin diseases	0.5	1.33	[1.25; 1.41]	1.36	[1.22; 1.52]	1.31	[1.22; 1.41]
11. Musculoskeletal diseases	0.6	1.14	[1.08; 1.20]	1.19	[1.09; 1.30]	1.12	[1.05; 1.19]
12. Genitourinary diseases	1.4	1.18	[1.14; 1.22]	1.21	[1.16; 1.27]	1.15	[1.10; 1.21]
13. Pregnancy, childbirth	0.0	1.60			[1.15; 2.23]	1.60	[1.15; 2.23]
14. Perinatal diseases	0.3	1.11	[1.03; 1.19]	1.10	[1.00; 1.22]	1.11	[0.99; 1.24]
15. Congenital malformations	0.3	1.29	[1.20; 1.39]	1.30	[1.18; 1.44]	1.28	[1.15; 1.42]
16. Ill-defined conditions ^b^	6.3	0.86	[0.85; 0.88]	0.83	[0.81; 0.85]	0.89	[0.87; 0.91]
17. Injury, poisoning ^c^	8.0	1.50	[1.47; 1.52]	1.67	[1.64; 1.71]	1.27	[1.25; 1.30]

'Avoidable' causes, < 65 years^1^	7.1	1.77	[1.74; 1.81]	1.83	[1.79; 1.86]	1.59	[1.53; 1.65]
Other causes, < 65 years^2^	13.6	1.32	[1.31; 1.34]	1.37	[1.35; 1.39]	1.24	[1.22; 1.27]

All causes	100.0	1.27	[1.26; 1.28]	1.33	[1.32; 1.34]	1.18	[1.17; 1.19]

FDep99: First component of a PCA on the *commune *scale of the variables: median income; % blue-collar workers; % high school graduates; and unemployment rate

% Total: percentage of the total mortality

SMR: standardized mortality ratio for the period 1997–2001.

SMRQ5/SMRQ1: ratio of the SMR of *communes *whose FDep99 index is higher than the fourth quintile and SMR of the *communes *whose FDep99 index is lower than the first quintile

^1^: Causes of death, linked to risk behaviours and targeted by primary prevention, for subjects aged less than 65 years; (lung, trachea and bronchus cancers (ICD10 Code: C32–C34), aerodigestive tract cancers (C00–C14), oesophagus cancer (C15), alcohol abuse (F10), chronic liver disease (K70, K73–K74), drug dependence (F11), AIDS (B20–B24), transport accidents (V01–V99), suicides (X60–X84) and homicides (X85–Y09))

^2^: All other causes for subjects aged less than 65

#### By gender

Mortality differentials for cancer were only observed for men, and were of smaller amplitude than for all-cause mortality. Specifically, the mortality differentials were more marked for men than women for mental disorders, injury and poisoning, respiratory diseases and digestive system diseases.

#### 'Avoidable' mortality

The 'avoidable' mortality, related to risk behaviours and targeted by primary prevention, for subjects aged less than 65 years, was much more strongly associated with the FDep99 index (+77% between the fifth and the first quintile), than the other causes for the same age group (+32%). The difference in the associations for 'avoidable' mortality and other causes was more marked for men than for women (Table 2).

## Discussion

The spatial deprivation index, 'FDep99', was calculated on the *commune *scale for all of mainland France. The component variables were well correlated with the index with respect to deprivation.

The association between the FDep99 index and mortality was positive and quasi-linear. The SMR was 24% higher in the *communes *of the most deprived quintile than those of the least deprived quintile. The association was observed between and within each of the geographic scales and in each urban unit category (UUC). With regard to the log-linear trends of the association on the *commune *scale, the between-UUC and between-*région *heterogeneities were not statistically significant. The association was positive for all the categories studied and was significantly stronger for subjects aged less than 65 years, men, and 'avoidable' mortality.

The amplitude and regularity of the association between mortality and the Townsend and Carstairs indices were lower.

The FDep99 index is not intended for investigating for a causal relationship between deprivation and mortality. The index is mainly designed for broad, routine description of health inequalities related to spatial disparities in socioeconomic level.

Moreover, despite the strong and regular association of the index with mortality, the index clearly does not totally explain the geographic heterogeneity of mortality in France. In particular, after adjustment for deprivation, the between-*région *heterogeneity remains high and statistically significant. This heterogeneity could be attributed to many other factors (environment, diet, lifestyle, etc.).

Because of confidentiality constraint, income data were not available for communes of less than 50 households. Those small *communes *were almost all in rural UUC and account for less than 5% of the rural UUC population. This population was likely to be very specific. However, given that the priority was to take income into account as a fundamental dimension of deprivation, those *communes *were excluded from the analysis.

### Deprivation index

Socioeconomic level may be associated with health at an 'individual' and/or 'contextual' level. In an ecological analysis, those two levels cannot be distinguished. However, some authors built their synthetic deprivation indices in order to approximate each subject's socioeconomic level, in particular by standardizing the components by age and gender [4,6]. The FDep99 index was not designed for that purpose and its components were therefore not age- or gender-standardized. Thus, the FDep99 index takes into account the potential influence of the population structure by age and gender on contextual socioeconomic level.

The construction of the FDep99 index is very similar to that of the most recent indices [4-9]. The FDep99 index is proposed in a pragmatic manner, based on the small-scale socioeconomic data available in France, and has been validated using non-exhaustive criteria. The constructed index cannot be used, as such, on other countries' data because it remains influenced by the choice of the variables available and their weightings, which were determined using French data. The proposed approach to index construction took account of the structural spatial socioeconomic specificities of a population, without any *a priori *link with general mortality.

When the whole of France was considered, the FDep99 index was not well correlated with the Townsend and Carstairs indices. More specifically, the FDep99 index was lower for urban categories than for rural categories, while the Townsend and Carstairs indices exhibited the opposite pattern. This difference is mainly due to the inclusion of variables related to car ownership and housing, whose practical meanings are dependant on the rural-urban gradient, in the definition of the Carstairs and Townsend indices. With the French data, those variables did not co-vary between-UUC according to the other deprivation components considered. Therefore, the variables were not included in the FDep99 index. Other studies have also shown that the Townsend and Carstairs indices are not necessarily consistent over a whole country, but rather over areas in which the degree of urbanicity is quite homogeneous [10,11,24,42]. That limitation does not seem to apply to the FDep99 index.

The correlations with the Townsend and Carstairs indices were higher for within-UUC correlations.

The weak correlations may also be a result of the approximations of the Townsend and Carstairs indices due to the French census variables available.

### Association between FDep99 index and mortality

The results showed the homogeneity of the association between the FDep99 index and all-cause mortality, with regard to the various degrees of urbanicity and the regions. This result, rarely observed [10,20,21,43], is consistent with the FDep99 index having a homogeneous meaning over the whole country.

In addition, the association between mortality and deprivation was observed on all the geographic scales and for most of the disease categories. Taking socioeconomic factors into account in ecological analyses thus appears necessary, irrespective of the scale considered. Moreover, deprivation is associated with health in general, despite the specifically stronger associations for certain diseases and the very diverse mechanisms involved [44,45].

The weaker association for the elderly observed in this study has already been reported [9,18,35]. This finding may be due to selection of the healthiest subjects in the lower social categories.

The association between deprivation and all-cause mortality for women and for children aged under 1 year was generally significantly positive, but the amplitude was smaller than for the other categories. The result, already reported in other studies [6,9,12,20,34-36], is likely to reflect the lower frequency of risk behaviours and occupational exposures in those population categories. With regards to female cancers, for which the association is almost nil, the generally non-positive association between socioeconomic level and breast cancer [46], and the negative gradient between smoking and socioeconomic level in women aged over 45 years [47] are noteworthy.

In this study, the mortality indicators and deprivation indices were considered cross-sectionally and defined over closed time periods. One of the limitations of this approach derives from the possibility that the influence of deprivation on a population, possibly giving rise to a higher mortality rate, is also exerted over a period preceding death. This is obvious for long term exposure differentials or subjects that have moved. Nevertheless, in most cases, the deprivation of a spatial unit at a given time reflects a long time course and common socioeconomic characteristics shared by the subjects living in, or moving into, that unit. However, it may be important to analyse associations between deprivation and mortality by including time lags between the two phenomena.

### Public health implications

As is the case in many other countries, the 'Profession and socioprofessional category' is the only individual variable characterising socioeconomic level in French death records. The category is only reliably reported for subjects aged between 25 and 55 years. Thus, another approach is needed for the analysis of socioeconomic mortality differentials, even if it is purely descriptive. The ecological approach, via the deprivation concept, has the advantage of being routinely feasible without incurring strong confidentiality constraints. In addition, deprivation reflects the socioeconomic disparities of the whole population, including subjects aged over 55 years.

## Conclusion

The deprivation index reflects a major part of spatial socioeconomic heterogeneity, in a homogeneous manner, over the whole country.

The strong and regular association of the index with mortality suggests that an ecological deprivation index built using a simple method, like that proposed herein, or constructed following broader consultation of the various observers of social inequalities, such as the UK Indicator of Multiple Deprivation [14] (IMD), should be adopted by health authorities in order to observe, analyse and manage spatial health inequalities.

## Competing interests

The authors declare that they have no competing interests.

## Authors' contributions

GR and DH were the principal investigators of the study. GR was in charge of the statistical modelling and analysis of data, contributed to the interpretation of the data and drafted the manuscript. DH provided statistical and epidemiological expertise and participated in the interpretation of the data. EJ contributed to the acquisition of the data on all-cause mortality and mortality by medical cause of death, and participated in the interpretation of the data. AF participated in the statistical analysis and in the interpretation of the data. All the authors revised the manuscript and have approved the final version.

## Pre-publication history

The pre-publication history for this paper can be accessed here:


